# What are the determinants of parametrial invasion in patients with early stage cervical cancer: A cross sectional study

**DOI:** 10.1016/j.amsu.2022.104020

**Published:** 2022-06-20

**Authors:** Niloufar Hoorshad, Narges Zamani, Shahrzad Sheikh Hasani, Amirhossein Poopak, Amirsina Sharifi

**Affiliations:** a Department of Obstetrics and Gynecology, Tehran University of Medical Sciences, Tehran, Iran; b Department of Gynecology Oncology, Vali-e-Asr Hospital, Tehran University of Medical Sciences, Tehran, Iran; c Cancer Research Center, Cancer Institute, Tehran University of Medical Sciences, Tehran, Iran; d Sina Trauma and Surgery Research Center, Tehran University of Medical Sciences, Tehran, Iran

**Keywords:** Cervical cancer, Early-stage, Parametrial invasion, Less invasive surgery

## Abstract

**Introduction:**

There was an increase in number of patients presented with early-stage cervical cancer (CC). Tumors with favorable pathological features might be candidates for less radical surgery.

**Methods:**

We retrospectively reviewed 700 patients with histologically confirmed CC between January 2011 and March 2020. Chi-square, Fisher's exact tests and multivariate logistic regression analysis were used to assess relations between parametrial involvement (PI) and clinic-pathological variables.

**Results:**

Total number of 132 patients with stage IA to IIA were eligible to participate. Squamous cell carcinoma was reported in 100 (75.8%) patients, adenocarcinoma and other tumor pathologies were found in 24(18.2%) and 8(6.1%), respectively. Considering the 2018 FIGO stage, 11 (8.4%) patients had IA, 111 (83%%) IB and 10 (7.6%) IIA. Nine patients (6.8%) had PI on permanent pathologic report. Univariate analysis demonstrated that following variables were statistically different between patients with and without PI: age ≥50, tumor size ≥ 3 cm, lower segment involvement, poorly differentiated pathology, deep stromal invasion, pelvic lymph node, lympho-vascular involvement and positive surgical margin (all p values < 0.05). Among these variables only tumor size ≥3 cm (OR: 2.1, 95% CI: 1.11–4.16, p value: 0.02), deep stromal invasion (OR: 2.2, 95% CI: 1.9–7.43, p value: 0.02) and positive surgical margin (OR: 5.1, 95% CI: 3.97–11.15, p value: 0.008) were independent risk factor of PI in multivariate analysis.

**Conclusions:**

Early stage CC might be surgically approached in a more conservative manner if patients have tumor size <3 cm and do not have deep stromal invasion in conization.

## Highlights

• The parametrium was resected, in order to remove occult disease at the time of extirpation of the primary cervical lesion.
• This study revealed, tumor size, deep stromal invasion and surgical margin were independent risk factors for parametrial invasion.
• Selected patients in early stages of cervical cancer are possible candidates to undergo a more conservative approach.

## Abbreviations

CC: cervical cancer
FIGO: International Federation of Gynecology and Obstetrics
PI: parametrium involvement
LVSI: lymphovascular space invasion
SPSS: Statistical Package of Social Science software
MRI: Magnetic Resonance Imaging

## 1 Introduction

Cervical cancer (CC) is the most leading cause of cancer death among females worldwide with approximately over 500000 new cases per year and 256700 deaths [1]. Public awareness of the disease and national screening programs has led to the increased number of patients being diagnosed in early stages (IA-IIA) even in developed countries [2]. These studies demonstrated that there was a growing pattern in both incidence and public knowledge about CC. Thus it is predicted that in following years there will be a jump in number of patients being diagnosed with early stages of CC [3].

The treatment of choice for early stage CC based on International Federation of Gynecology and Obstetrics (FIGO) guide line is radical hysterectomy with pelvic lymph node dissection [4]. Radical trachelectomy has been introduced as a possible option to preserve fertility in selected women [5]. The most common type of radical hysterectomy, known as Wertheim procedure, consists of removal of the uterus, upper vagina, uterosacral ligaments, and parametrium [6]. It has been shown that resection of parametrium is associated with significant morbidity in up to 38% of patients including bladder dysfunction, sexual dysfunction (vaginal dryness), and rectal dysmotility [7]. The main reason of these morbidities is the damage made to autonomic nerve fibers, which travel in the parametrium and control the bladder, bowel, and sexual function [8]. Historically, the parametrium was supposed to be resected, in order to remove occult disease at the time of extirpation of the primary cervical lesion. The logic behind this approach was the fact that parametrium received lymphatic drainage of cervix [9]. Therefore, an optimal surgical resection of the tumor which obligates as low as possible postoperative complications should be pursued.

The objective of this study was to identify risk factors of parametrium involvement (PI) in early stage CC.

## 2 Methods

We retrospectively analyzed medical records of 700 patients with histologically confirmed CC between January 2011 and March 2020 at Imam Khomeini Hospital Complex. The institutional board of Tehran University of Medical Sciences approved the study protocol. All patients in stage IA to IIA (2018 FIGO staging system)whom underwent radical hysterectomy(type III) and pelvic lymphadenectomy, were selected [10,11]. Patients with the following characteristics were excluded: Preoperative FIGO stage greater than IIA. Any surgery that does not include parametrectomy, any neoadjuvant therapy and patients with missing histologic data.

Following information were retrieved from patient medical records: age, tumor stage, tumor histology, pelvic and paraaortic lymph status, total number of lymph node harvested in surgery, tumor size, lymphovascular space invasion (LVSI), depth of stromal invasion, surgical margin, vaginal lower segment involvement status and PI. Pathological reports were checked by two independent attending gynecologic pathologists blinded to the aims of the study. PI was defined as the presence of tumor cells in or beyond the parametrial vessels [12]. Deep stromal invasion was defined as either >10 mm cervical stromal invasion or its involvement more than two-thirds of cervical thickness.

Categorical variables are shown as frequency (%) and continuous variables are shown as mean ± standard deviation. Categorical variables were compared using the chi-squared test. An independent student t-test was used to compare means between the two groups. All analyses were performed by the two-sided method using Statistical Package of Social Science software (SPSS version 22; SPSS, Inc., Chicago, IL), and the p-value of <0.05 was set as statistically significant.

This research was carried out in compliance with the Helsinki Declaration and was approved by the ethical committee at Tehran University of Medical Sciences (IR.TUMS.IKHC.REC.1398.125).

This study is fully compliant with the STROCSS criteria www.strocssguideline.com [13].

## 3 Results

Total number of 132 patients were eligible to participate in the study. The mean ± SD of age for entire population was 47.08 ± 10.98 years. Twenty-seven percent (36 patients) of entire study population were nulliparous. The most common pathology of cervical cancer was squamous cell carcinoma reported in 100 (75.8%) patients; adenocarcinoma and other tumor pathology were found in 24(18.2%) and 8(6.1%), respectively. The histologic grade of the tumor was poorly differentiated, moderately differentiated and well differentiated in 30(22.7%), 65(49.2%) and 37(28%) patients, respectively. Considering the FIGO stage, 10 (7.6%) patients had IA1, 1 (0.8%) IA2, 20 (15.2%) IB1, 61 (46.2%) IB2, 30 (22.7%) IB3, 10 (7.6%) IIA, respectively.

Nine patients (6.8%) had PI on permanent pathologic reports. Patients with PI and those without were compared based on demographic characteristics and permanent pathologic reports which is shown in Table 1. Patients were divided into those <50 and those ≥50 years old. Patients with PI were significantly older (7/9 (77%) Vs 50/123 (40%), p value: 0.03). Patients with PI had significantly larger tumors as it was shown that all of the patients with PI had tumor with greatest dimension > 3 cm (p value: 0.003) and 7 out of nine had tumor > 4 cm (p value = 0.001). Lower segment involvement was found in 4 out of 9 (44%) patient with PI and 21 out of 123 patients (17%) without it (p value: 0.05). Patients with positive PI had higher frequency of tumors with poorly differentiated pathology (5/9 (55%) Vs 25/123(20%), p value: 0.04).

**Table 1:** Permanent pathologic report in study population based parametrial involvement.

Variable		Parametrial Involvement (n = 9)	No parametrial Involvement (123)	P value
Age, (Mean ± SD)		52 ± 8.1	46.72 ± 11.1	0.04
parous	Nulliparous, n(%)	1 (11%)	35(28%)	0.44
	Multiparous n(%)	8(88%)	88(71%)	
Age group, n(%)	<50	2(22%)	73(59%)	0.03
	≥50	7(77%)	50(41%)	
Pathologic type, n(%)	Squamous	6(66%)	94(77%)	0.74
	Adenocarcinoma	2(22%)	22(18%)	
	Others	1(11%)	7(5%)	
FIGO stage, n(%)	IA1	0(0%)	10(8%)	<0.0001
	IA2	0(0%)	1(1%)	
	IB1	0(0%)	20(16%)	
	IB2	2(22%)	59(48%)	
	IB3	5(55%)	25(20%)	
	IIA	2(2%)	8(6%)	
Tumor differentiation, n(%)	Well differentiated	1(11%)	36(29%)	0.04
	Moderately differentiated	3(33%)	62(50%)	
	Poorly differentiated	5(55%)	25(20)	
Deep stromal invasion, n(%)	Yes	8(88%)	58(47%)	0.03
	No	1(11%)	65(52%)	
Greatest tumor Size, n(%)	≥2	9(100%)	92(75%)	0.11
	≥3	9(100%)	59(48%)	0.003
	≥4	7(77%)	27(22%)	0.001
Vaginal involvement, n(%)	Yes	2(22%)	10(8%)	0.19
	No	9(77%)	113(91%)	
Lower segment involvement, n(%)	Yes	4 (44%)	21(17%)	0.05
	No	5(55%)	102(82%)	
Lympho-vascular invasion, n(%)	Yes	7(77%)	53(43%)	0.04
	No	2(22%)	70(56%)	
Pelvic lymph node involvement, n(%)	Yes	5(55%)	12(15%)	0.01
	No	4(44%)	111(90)	
Para-aortic lymph node involvement, n(%)		0(0%)	0(0%)	–
Lymph node harvested, (Mean ± SD)		18.2 ± 5.03	18.86 ± 9.24	0.85
Surgical margin Involvement, n(%)	Yes	4(44%)	3(2.5%)	<0.0001
	No	5(55%)	120(97.5)	

Patients with PI tended to have deep stromal invasion (8/9 (88%) Vs 58/76 (47%), p value: 0.03). Comparing patients with and without PI, vaginal (2/9 (22%) Vs 10/123 (8%), p value: 0.19) was higher in those patients with PI. We assumed that patients with PI would have higher frequency of pelvic and para-aortic lymph node involvement. Pelvic lymph node involvement was seen in 5 (55%) patients with PI and 19(15%) patients of not-PI (p value: 0.01). None of the patient had para-aortic lymph node involvement. Mean total number of lymph node harvested from surgery was 18.2 ± 5.03 in PI group and 18.86 ± 9.24 in not-involved group (p value: 0.85). LVSI was present in 7 (77%) patients with PI and 53 (43%) patients without PI (p value: 0.04). Positive surgical margin was found in 4 (44%) patients with and 3 (2.5%) patients without PI, respectively (p value < 0.0001).

After multivariate analysis of baseline characteristics and permanent pathologic report, tumor size ≥3 cm (OR: 2.1, 95% CI: 1.11–4.16, p value: 0.02), deep stromal invasion (OR: 2.2, 95% CI: 1.9–7.43, p value: 0.02) and positive surgical margin (OR: 5.1, 95% CI: 3.97–11.15, p value: 0.008) were introduced as independent risk factors for PI. Other variables which were significantly different between two groups in univariate analysis did not show significance in multivariate study (Table 2).

**Table 2:** Multivariate analysis for predictive factors associated with parametrial involvement.

Variable	Parametrial involvement		
	OR	CI (95%)	P value
Age >50	8.3	0.54–16.73	0.74
Deep stromal invasion	2.2	1.9–7.43	0.02
Tumor size >3 cm	2.1	1.11–4.16	0.02
Lower segment involvement	0.38	0.04–50.32	0.83
Lympho-vascular invasion	0.39	0.19–11.55	0.70
Pelvic lymph node involvement	1.06	0.23–35.80	0.44
Positive surgical margin	5.1	3.97–11.15	0.008

## 4 Discussion

We found PI in nearly 7% of the patients. Age ≥50 years old, being poorly differentiated, tumor size ≥ 3 cm, having deep stromal invasion, pelvic lymph node involvement, LVSI, and incomplete tumor resection were significantly more frequent in patients with PI but only the three factors of tumor size ≥ 3 cm, deep stromal invasion and positive surgical margin due to incomplete tumor resection were independently associated with PI in multivariate analysis.

English literature on the factors associated with PI is quiet controversial as the optimal resection margin with the least post-operative complication rate in patients undergoing surgery for early stage CC is not clearly addressed. The reason behind this discrepancy is the fact that it was not possible to identify predictable patterns of dissemination with concomitant invasion of the medial and lateral parametria [14]. There are reports that PI happens through direct extension in 37%, by lymph node metastases in 59% and LVSI in 52% of cases [15]. These findings explain why it is difficult to reduce the extent of the surgical resection without leaving residual tumoral tissue behind. So there are two strategies to reduce these complications through either modification of surgical techniques or design selection criteria to find patients who would benefit from radical surgery the most.

An outstanding study on patients with early-stage (IA2–IIA) CC from 10 French university hospitals was executed by Dabi et al. [12]. Out of the 263 patients included, 28 (10.6%) had PI. In this study, factors significantly associated with PI on multivariate analysis were: age>65 years, tumor>30 mm in diameter measured by (Magnetic Resonance Imaging) MRI and LVSI. Among the 235 patients with negative pelvic lymph nodes, PI was seen in only 7.6% compared with 30.8% of those with positive pelvic nodes (p value < 0.001). Baiocchi et al. [7], analyzed a series of 345 patients with stage IA2 to IB2 cervical cancer whom underwent radical surgery. Sixteen (4.6%) patients had PI in their series. The presence of perineural invasion (p value = 0.003), tumor size >2 cm (p value = 0.044), depth of invasion >10 mm (p value = 0.004), the presence of LVSI (p value < 0.001), and lymph node metastasis (p value < 0.001) were related to PI. However, only LVSI and lymph node metastasis remained significant risk factors for PI in the multivariate analysis. Authors concluded that patients with tumors ≤2 cm and those who lack LVSI are unlikely to have PI, unless lymph node metastasis or deep stromal invasion is present. In the study performed by Boyraz et al. [16], 126 patients with early stage CC were investigated and PI was detected in 41 (32.5%). Univariate analysis showed that deep cervical stromal invasion, LVSI, tumor size >2 cm and lymph node metastasis were significantly associated with PI. However, multivariate logistic regression analysis identified the independent risk factors associated with PI as LVSI (OR 8.93, 95% CI 1.1–73.5, p = 0.042) and lymph node metastasis (OR 8.8, 95% CI 1.5–9.3, p = 0.004). Our findings are along with these studies and all of them emphasize on the fact that a more conservative approach is warranted, in selected patients.

Another way to predict PI is through nomograms. Nomograms are designed to assess an individual probability of a certain event with validated indications [17]. Kong et al. [18]., described a nomogram for patients with stage IB using diameter-based tumor volume and disruption of the cervical stromal ring on MRI, serum squamous cell carcinoma antigen level, and menopausal status to predict PI preoperatively. They claimed that the concordance index of the nomogram was 0.940 (95% CI, 0.908–0.967), and it has revealed good agreement between the observed probabilities and nomogram- Benoit et al. [19]., used two prospective multicentric databases—SENTICOL I and II—to develop a nomogram to predict PI in patients with IA to IIA1 CC. They found sentinel lymph node status, LVSI, deep stromal invasion and tumor size were significantly associated with PI and were included in their nomogram. They announced their predictive model had an area under the curve of 0.92 (confidence interval 95% = 0.86–0.98) and presented a good calibration.

This study has an innate limitation of being conducted as a retrospective study so further studies with a more accurate methodology are warranted. Finally, the small sample size of the study made it possible to reduce the study power and therefore conduct further studies such as multicenter research with larger sample size.

## 5 Conclusion

In conclusion, this study shows the association of parametrial involvement with tumor size, grade of tumor, lymph node status, invasion depth, surgical margin involvement, and LVSI. Our results suggest that selected patients in early stages of CC with tumor size <3 cm and without deep stromal invasion are possible candidates to undergo a more conservative approach especially in centers where do not have any specialists.

## Please state any conflicts of interest

Nothing to declare.

## Please state any sources of funding for your research

Nothing to declare.

## Ethical approval

This research was carried out in compliance with the Helsinki Declaration and was approved by the ethical committee at Tehran University of Medical Sciences (IR.TUMS.IKHC.REC.1398.125).

## Consent

All the patients signed the informed consent form. A copy of the written consent is available for review by the Editor-in-Chief of this journal on request.

## Author contribution

1 N.H.: collecting data
2 N.Z.: writing and editing the final manuscript, corresponding
3 Sh.Sh.: collecting data
4 A.P.: data analysis
5 A.Sh.: editing the article

## Registration of research studies

1. Name of the registry: Iran National Committee for Ethics in Biomedical Research
2. Unique Identifying number or registration ID: IR.TUMS.IKHC.REC.1398.125
3. Hyperlink to your specific registration (must be publicly accessible and will be checked): IR.TUMS.IKHC.REC.1398.125).

## Guarantor

Narges Zamani.

## Ethics approval and consent to participate

This research was carried out in compliance with the Helsinki Declaration and was approved by the ethical committee at Tehran University of Medical Sciences (IR.TUMS.IKHC.REC.1398.125).

## Consent for publication

All the patients signed the informed consent form. A copy of the written consent is available for review by the Editor-in-Chief of this journal on request.

## Availability of data and materials

The datasets used and/or analyzed during the current study are available from the corresponding author on reasonable request.

## Funding

This research did not receive any specific grant from funding agencies in the public, commercial, or not-for-profit sectors all authors have read and approved the manuscript.

## Declaration of competing interest

The authors declare that they have no competing interests.
